# Psychological interventions for individuals affected by Huntington’s disease: effects on depression, anxiety, and emotional well-being

**DOI:** 10.1007/s12687-026-00923-6

**Published:** 2026-09-02

**Authors:** Andrada Ciucă, Ștefan Trif, Simona Samoilă, Mădălina Radu, Francisco Iruela, Mónica Rodríguez Lopera, César Aguilera Vega, Ruth Blanco, Filipa Júlio, Astri Arnesen, Ramona Moldovan

**Affiliations:** 1 Department of Psychology, Babeş-Bolyai University, Cluj-Napoca, Romania; 2 Valencian HD Association (Asociación Valenciana de Enfermedad de Huntington), Alacant, Spain; 3 Balearic Huntington Association (Asociación Balear de Corea de Huntington), Mallorca, Palma, Spain; 4 Spanish HD Association (Asociación Corea de Huntington Española), Madrid, Spain; 5 Foundation for Biomedical Research of the Ramón y Cajal University Hospital, Madrid, Spain; 6 European Huntington Association, Moerbeke-Waas, Belgium; 7 Division of Evolution, Infection and Genomic Sciences, School of Biological Sciences, University of Manchester, Manchester, M13 9PT UK; 8 Manchester Center for Genomic Medicine, St. Mary’s Hospital, Manchester University NHS Foundation Trust, Manchester, M13 9WL UK

## Abstract

**Introduction:**

The literature provides limited evidence on the effectiveness of psychological interventions for individuals living with Huntington’s Disease (HD). This study aims to examine the role of psychological support in improving the well-being of individuals and families affected by HD.

**Methods:**

The study methodology builds on previous work conducted by the European Huntington Association (EHA). The study includes individuals across three categories: individuals at risk of HD, premanifest HD, or with an HD-negative genetic test. Eligible participants were offered eight online individual cognitive behavioural therapy (CBT) sessions followed by two group sessions. Assessments were performed at pre-intervention, after the individual sessions and after the group sessions. The primary outcomes were anxiety, depression, positive and negative affect, and quality of life.

**Results:**

Twenty-one individuals participated in the study, of whom 33% (n = 7) had an HD-negative genetic test, 29% (n = 6) were at risk, and 38% (n = 8) had premanifest HD; the cohort was 57% women (n = 12). Analyses showed significant reductions in depression and anxiety, with medium to large effect sizes. Negative affect also decreased significantly with a large effect, while positive affect showed a non-significant trend toward improvement. Quality-of-life outcomes (physical and mental health components) did not change significantly. Overall, the strongest effects were observed for mood-related outcomes (depression, anxiety, and negative affect).

**Conclusion:**

Our data shows that a short-term CBT intervention led to improvements in anxiety, depression and negative affect. These findings enhance the evidence base available supporting the efficacy of psychological interventions that may be beneficial for individuals affected by HD. The article also outlines key lessons regarding intervention tailoring and study design to inform future research exploring the efficacy of psychological interventions for this population.

## Introduction

Huntington’s disease (HD) is a hereditary neurodegenerative disorder caused by a CAG trinucleotide repeat expansion in the huntingtin gene (HTT), which leads to the production of abnormal huntingtin protein. Individuals with symptomatic HD typically experience progressive decline in three core domains: motor function (e.g., chorea, dystonia), cognition (e.g., impaired decision-making, concentration), and behavioural symptoms (e.g., depression, mood swings, irritability) (Bates et al. 2014).

The motor, cognitive and behavioural changes related to HD are generally thought to contribute to the burden and stigma often experienced by families impacted by HD (Boileau et al. 2020; Wexler 2010). The severity and complexity of HD symptomatology is also thought to significantly impact mental health (Gunn et al. 2023) and quality of life of individuals impacted by HD (Pearl et al. 2023). Additionally, HD is known to exert a substantial psychological impact on extended family members, namely individuals at risk, those with premanifest HD, as well as individuals with a negative genetic test result (Tillerås et al. 2020; Spiers et al. 2020).

Individuals at risk for HD or in the premanifest stage often experience elevated levels of depression, anxiety, and emotional distress driven by uncertainty surrounding their genetic status and the anticipation of disease onset (Crozier et al. 2014; Dale and van Duijn 2015). While an estimated 17.4% (Baig et al. 2016) to 24% (Laccone et al. 1999; Harper 2000) of at-risk individuals worldwide choose to undergo predictive genetic testing, pre-test levels of anxiety and depression have been shown to be stronger predictors of post-test psychological distress than the test result itself (Decruyenaere et al. 2003; Spiers et al. 2020). This highlights not only the importance of pre-test and post-test genetic counselling, but also suggests that the at-risk period represents a critical moment for psychological interventions. Moreover, post-test distress remains a significant concern. A systematic review by Crozier et al. (2014) found that distress levels were comparable between individuals who tested positive and those who tested negative, with fluctuations over time, underscoring the complex and enduring psychological impact of predictive testing. Individuals who receive a positive test result initially reported emotional responses such as “shock, denial and anger, regret at taking the test, feeling that life is over, distress at anticipated life changes, worries about future relationships, fears of discrimination and loneliness” (Theed et al. 2017). These experiences may significantly impact quality of life, potentially to a greater extent than motor or cognitive symptoms in the early stages of the disease (Ho et al. 2009). However, individuals who receive a negative test may also face considerable psychological challenges adapting to the result, including pressure to achieve something extraordinary in their lives or feelings of guilt, which can lead to sadness or clinical depression (Winnberg et al. 2018). Given the substantial psychological burden experienced by individuals across the HD spectrum - whether at-risk, gene-positive or gene-negative - effective management of psychiatric symptoms is critical. Up to 98% of individuals report psychiatric complaints at some point during the course of the disease, either transiently or continuously, with different stages of HD presenting different challenges (Mühlbӓck et al. 2023). Despite the historical reliance on pharmacological approaches, antidepressants often fall short in clinical efficacy. In general populations, medication-based treatments for psychiatric disorders are only effective in about one-third of cases, leading to a significant proportion of therapy-resistant individuals (Pelgrim et al. 2024; van Westrhenen et al. 2020). In the case of HD, this challenge may be further compounded by the disease-specific neurobiological mechanisms. Emerging evidence suggests that HD-related depression involves distinct pathways compared to gene-negative populations, potentially complicating the assessment and efficacy of traditional antidepressants (Du et al. 2013; Zadegan et al. 2024a). For example, a 2022 retrospective study of motor-manifest HD revealed no significant improvement in depressive or anxiety symptoms among antidepressant users compared to non-users, even after adjusting for disease progression (Ogilvie et al. 2022). Beyond their limited effectiveness, antidepressants are frequently associated with side effects (e.g., nausea, fatigue) which may further complicate the existing drug regimens of individuals with HD and may worsen symptoms like apathy or motor impairment (van Duijn 2017). Given these limitations, individuals with HD have expressed a preference for psychological interventions as a more empowering approach to managing the impact of mental health challenges related to an HD diagnosis. Research has shown that psychological therapy is perceived as a means of actively addressing these challenges, offering a constructive complement to pharmacological treatments (Theed et al. 2017).

Despite growing recognition of these challenges, empirical evidence regarding the efficacy of psychological interventions in HD remains scarce. A recent study (Pires et al. 2024) highlighted significant concerns among individuals living with HD, including fragmented care, geographic inequalities in access to services, and unmet complex needs. To date, only a small number of studies have evaluated psychological interventions in this population. Two studies reported significant improvement in depression, anxiety and coping skills following cognitive and behavioural psychological interventions (CBT) in individuals with HD (A’Campo et al. 2012; Silver 2003). A narrative therapy support group showed reduced feelings of loneliness and improved coping strategies among individuals tested positive for HD or at risk (Stopford et al. 2019). Similar interventions have also shown reductions in anxiety and depression among individuals with a negative genetic test result (MacLeod et al. 2018), as well as an overall positive impact for individuals who tested positive (Spiers et al. 2020). A review summarizing available psychological interventions for HD identified nine studies, of which one included both manifest and premanifest participants, two included only premanifest participants, and six included manifest participants (Zarotti et al. 2020). The review, which encompassed a total of 66 participants, concluded that the research in this area remains limited in both methodological rigor and scope of clinical outcomes, and therefore no specific psychological therapy can be yet recommended. To our knowledge, this is the first study to address the needs of all three groups—at-risk, premanifest, and negative-test individuals—within a single intervention framework.

This study builds upon and extends the work of the Moving Forward project funded by the European Huntington Association (EHA). This is a transnational and multilingual initiative initiated in 2020 which was designed to provide tailored information and support to the younger generations of families impacted by HD. An online survey carried out by EHA highlighted a substantial need for psychological support among individuals at risk of HD and individuals with premanifest HD across Europe (Júlio et al. 2021). In response to these findings, in 2023, the EHA Moving Forward team designed an online psychological support service together with the Spanish Huntington’s Disease Association and conducted a pilot study involving individuals impacted by HD in Spain. The study methodology built on prior work conducted by the European Huntington Association. Sixteen participants at risk of HD and with premanifest HD were recruited through the Spanish HD Association (ACHE) and were offered a structured psychological intervention delivered over a six-month period (January–July 2023). All eligible participants were offered eight individual online sessions, followed by two online group sessions and one face-to-face group session. The intervention was grounded in a systemic therapy framework and tailored to participants’ individual needs. All individual sessions were delivered by a clinical psychologist. Outcome assessments were conducted by two neuropsychologists with expertise in Huntington’s disease who were not involved in delivering the intervention. Assessments were performed at two timepoints: pre-intervention (baseline; one week prior to the start of the individual sessions) and post-intervention (one week following completion of the group sessions). The primary outcomes included depression and anxiety, while secondary outcomes included quality of life and coping skills. Standardized measures included the Overall Anxiety Severity and Impairment Scale (OASIS; Osma et al. 2019), the Overall Depression Severity and Impairment Scale (ODSIS; Osma et al. 2019), the Goldberg Anxiety and Depression Scale (GADS; Reivan-Ortiz et al. 2019), the Coping Strategies Questionnaire (CAE; Sandín and Chorot 2003), and the Quality-of-Life Assessment Scale (GENCAT; Verdugo et al. 2009).

The promising results of the pilot study provided the foundation for the present study, which refined and extended the approach. Specifically, the goal of the current study is to evaluate the impact of a CBT-based psychological intervention on depression, anxiety, positive and negative affect and quality of life in individuals at risk of HD, individuals with premanifest HD, or with a negative genetic test result.

## Methods

### Study design

The cognitive-behavioural intervention (CBT) was tailored to address the unique psychological challenges faced by individuals at risk of HD, premanifest HD, or with an HD-negative genetic test, such as depression, anxiety, and quality of life. Assessments were performed at three timepoints: pre-intervention (T0), post individual sessions (T1) and post group sessions (T2). Participants were recruited between September 2024 and March 2025 and group sessions were organised in September 2025. All eligible participants were offered eight individual and two group psychotherapy sessions. Participants were offered the option of online or in person sessions; all participants opted for online sessions. All individual sessions were delivered by a registered clinical psychologist, with specialised training in CBT (SS); group sessions were co-facilitated by two registered clinical psychologists trained in CBT (SS, MR). Ethical approval for the study protocol and procedure was granted by Babeș-Bolyai University’s Ethics Committee. Written consent was obtained from all participants.

### Participants

A total of 21 participants were eligible for the study and were offered psychological support. Inclusion criteria consisted of individuals over 18 years of age and being in one of the three categories: (1) at risk of HD (without genetic testing); (2) with premanifest HD (positive genetic test but no clinical symptoms); and (3) with a negative genetic test for HD. Individuals were recruited through multiple channels to ensure a diverse and representative sample. These included (1) the Romanian Registry for Huntington’s Disease; (2) the Romanian Huntington Association’s social media platforms; and (3) other professional networks involved in supporting HD families. Potential participants were presented with the overview of the study and had the opportunity to ask questions; those interested were sent a copy of the Patient Information Sheet and Informed Consent form. Potential participants were given approximately a week to read and ask further information about the study.

### The psychotherapeutic intervention

Based on a literature review of evidence-based psychological interventions, the psychotherapeutic support provided was informed by cognitive behavioural therapy (CBT), as it was thought to be the most appropriate approach for addressing key mental health challenges related to HD, including anxiety and depression. The eight individual sessions were delivered weekly, online, with each session lasting approximately one hour. The individual session phase of the intervention lasted for about 2 months for each participant. The sessions addressed emotional distress, anxiety, depression, coping skills, and quality of life and were tailored to each individual’s personal context. In detail, common contexts addressed were around family-planning or career decisions, carer roles, balancing work and family care, grief and loss related to HD, and individuals’ personal relationship with predictive testing and coping regardless of genetic status or test choice. Therapeutic strategies included psychoeducation, cognitive restructuring, behavioural activation, coping skills training, relaxation and mindfulness, and development of problem-solving skills. Mindfulness and relaxation were integrated alongside CBT techniques to target relevant emotional dysregulation; mindfulness to increase present‑moment tolerance of uncertainty and reduce rumination and relaxation to reduce physiological arousal and improve sleep. To ensure consistency and provide professional support, monthly supervision and mentoring sessions were organised for the clinical psychologist delivering the intervention. Following the completion of the individual sessions, participants were offered two group sessions, each lasting approximately two hours; the first was conducted online and the second took place in person, with about one month in between. The sessions aimed to promote adaptive coping strategies for managing anxiety, strengthen awareness and use of internal resilience resource and external sources of supports, as well as foster a sense of connection and belonging among participants. The strategies included experiential exercises focused on relaxation using guided imagery, exploration of personal values and values-based actions, and creating connection and mutual support within the group. The overall goal was to create a safe, collaborative environment that encouraged openness, shared understanding, and the development of effective psychological coping mechanisms in the context of HD.

### Assessment and timepoints

The assessments were conducted before and after the individual and group sessions, by a clinical psychologist (ST) who was not involved in delivering the psychotherapeutic intervention. The first assessment (T0) was conducted to collect baseline data after participants provided written informed consent to participate in the study. Following completion of the eight individual online sessions, participants underwent the second assessment (T1) to evaluate changes in psychological outcomes. After completion of the group sessions, a third (T2) assessment was conducted. All assessments were carried out virtually via Zoom, where the assessor guided participants through the questionnaires in real-time, ensuring clarity, minimising errors and recording the answers in a digital format.

### Questionnaires

#### Patient health questionnaire (PHQ9

Kroenke et al. 2001) is a widely used self-report measure of depression severity. It consists of nine items assessing depressive symptoms, rated on a 4-point Likert scale ranging from 0 (not at all) to 3 (nearly every day).

#### Generalized anxiety disorder (GAD7

Spitzer et al. 2006) is a widely used self-report questionnaire for assessing severity of anxiety. It includes seven items rated on a 4-point Likert scale ranging from 0 (not at all) to 3 (nearly every day).

#### Positive and negative affect schedule (PANAS

Watson et al. 1988) is a measure of mood, comprising two relatively independent dimensions - Positive Affect (e, g., enthusiasm, excitement), and Negative Affect (e.g., anger, fear). It is suitable for use in both clinical and non-clinical populations and contains 20 items to be rated on a 5-point Likert scale ranging from 1 (very slightly or not at all) to 5 (extremely).

#### Goldberg anxiety and depression scale (GADS

Reivan-Ortiz et al. 2019) is a self-report measure assessing symptoms of anxiety and depression. It contains 18 items to be rated on a 6-point Likert scale from 0 (not at all) to 5 (very much).

#### The 12-item short form health survey (SF-12

Ware et al. 1996) is a self-reported outcome measure assessing the impact of health on everyday functioning. It is often used as a health-related quality of life measure (HRQoL).

### Statistical analysis

Statistical analyses were conducted to evaluate the impact of the cognitive-behavioural therapy (CBT) intervention on psychological outcomes across the assessment time points. The primary outcomes were anxiety and depression, while the secondary outcomes included quality of life and affect. Descriptive statistics were used to summarise participant demographics and baseline characteristics (see Table 1) as well as outcome variables (see Table 2). Both parametric (i.e. paired sample t-test) and non-parametric (i.e. Wilcoxon signed-rank test) analyses were performed to assess changes in scores from pre- to post- intervention. This dual analytic approach was adopted to enhance the robustness of the findings, account for the small sample size and potential outliers, and facilitate comparability with previous HD research. All statistical analyses were conducted using SPSS, with significance set at *p* < 0.05. For t-test, effect sizes (Cohen’s d) were calculated to quantify the magnitude of changes, with values of 0.2, 0.5, and 0.8 interpreted as small, medium, and large effects, respectively. For the Wilcoxon signed-rank test, effect sizes were reported as *r*, with values of 0.1, 0.3, and 0.5 interpreted as small, medium, and large effects, respectively.

## Results

### Participant demographics

Twenty-one participants were enrolled, with a mean age of 37.19 years (SD = 8.16) and 57.14% women. Participants were categorized as at-risk (*n* = 6, 28.6%), premanifest HD (*n* = 8, 38.1%), or negative genetic test (*n* = 7, 33.3%). All participants completed the pre intervention assessments. Four participants dropped out of the study, one due to maternity leave, and three for unknown/undisclosed reasons. In the systemic therapy group, 16 participants were enrolled, with a mean age of 34.62 years (SD = 9.71), of which 75% were women. Participants were at-risk (43.7%) or premanifest HD (56.3%). Details on the participants’ demographics from both groups can be found in Table 1.

**Table 1:** Participant demographics

	CBT		Systemic therapy	
Characteristics	Total, *N* = 21		Total, *N* = 16	
Age (years)				
Mean ± SD	37.19 ± 8.16		34.62 ± 9.71	
Gender, n (%)				
Men	9	42.86%	4	25%
Women	12	57.14%	12	75%
Study category, n (%)				
Risk	6	28.6%	7	43.7%
Negative	7	33.3%	n/a	
Positive	8	38.1%	9	56.3%
Ethnicity, n (%)				
Romanian	20	95.3%	1	6.2%
Hungarian	1	4.7%	n/a	
Spanish			15	93.8%
Education, n (%)				
High School	5	23.8%	6	37.4%
University	16	76.2%	10	62.6%
Occupation, n (%)				
Employed	14	66.7%	13	81.2%
Unemployed	7	33.3%	3	18.8%
Area, n (%)				
Urban	14	66.7%	13	81.2%
Rural	7	33.3%	3	18.8%

*n/a* not applicable

Descriptive statistics on all primary and secondary outcomes are included in Table 2.

**Table 2:** Primary and secondary outcomes

Construct		CBT			Systemic therapy	
	Instrument/ Subscale	T0 *n* = 21	T1 *n* = 17	T2 *n* = 9	T0 *n* = 16	T1 *n* = 16
Depression	PHQ-9	8.15 ± 5.95	5.94 ± 5.44	4.11 ± 3.66	n/a	
	GADS - Depression	3.57 ± 2.31	2.47 ± 2.55	1.89 ± 1.83	2.25 ± 2.21	1.19 ± 1.28
Anxiety	GAD-7	5.29 ± 3.47	5.65 ± 5.33	3.00 ± 2.35	n/a	
	GADS - Anxiety	4.71 ± 2.03	3.76 ± 2.49	3.67 ± 2.29	4.37 ± 1.63	2.50 ± 1.93
Positive Affect	PANAS - Positive	35.90 ± 4.99	33.29 ± 5.51	35.78 ± 4.24	n/a	
Negative Affect	PANAS - Negative	23.76 ± 6.33	21.88 ± 9.37	17.22 ± 5.65	n/a	
Physical Health (QoL)	SF-12 PCS	50 ± 10.23	51 ± 8.27	54.24 ± 5.60	n/a	
Mental Health (QoL)	SF-12 MCS	42 ± 9.62	46 ± 10.67	45.22 ± 12.85	n/a	

*PHQ-9* (Patient Health Questionnaire-9), *GADS* (Goldberg Anxiety and Depression Scale), *GAD-7* (Generalized Anxiety Disorder-7), *PANAS* (Positive and Negative Affect Schedule), *SF-12* (Short Form Health Survey-12) - Physical (*PCS*) and Mental (*MCS*) components, *QoL* (Quality of Life), *n/a* not applicable

### Statistical analysis

Paired-sample t-tests and Wilcoxon signed-rank tests were conducted to examine differences in outcome scores between pre- and post- individual sessions (T0-T1) and between pre-intervention and post-group sessions (T0-T2). Three participants were considered outliers and, although they completed all sessions, their data were excluded from the final analysis. One participant was excluded because all questionnaire scores were null thorough the study. A second participant was excluded following an exploratory analysis (stem-and-leaf plot) which showed that their difference in scores for anxiety and depression is a statistical outlier (-13 and -12). The third participant was excluded because, during the study, they were offered a neurological consultation that confirmed HD symptoms, after which they initiated neurological and psychiatric treatment. Table 3 presents all statistical coefficients for comparisons (t-test, Wilcoxon test) as well as effect sizes (d and r).

**Table 3:** Statistical coefficients

Construct		CBT study (T0-T1)		CBT study (T0-T2)		Systemic therapy study (T0-T1)	
	Instrument/ Subscale	Paired T-test	Wilcoxon	Paired T-test	Wilcoxon	Paired T-test	Wilcoxon
Depression	PHQ-9	t = 2.645 *p* = 0.020* d = 0.707	Z= -2.237 *p* = 0.025* *r* = 0.646	t= -2.740 *p* = 0.025* d = 0.750	Z = 2.398 *p* = 0.016* *r* = 0.906	n/a	
	GADS - Depression Subscale	t = 2.500 *p* = 0.027* d = 0.668	Z= -2.158 *p* = 0.031* *r* = 0.599	t = 0.937 *p* = 0.376 d = 0.312	Z= -0.638 *p* = 0.523 *r* = 0.22	t = 2.075 *p* = 0.056 d = 0.519	Z= -2.064 *p* = 0.039* *r* = 0.365
Anxiety	GAD-7	t = 2.113 *p* = 0.050* d = 0.564	Z= -1.897 *p* = 0.050* *r* = 0.526	t = 4.459 *p* = 0.002* d = 1.486	Z= -2.375 *p* = 0.018* *r* = 0.898	n/a	
	GADS - Anxiety Subscale	t = 3.097 *p* = 0.008* d = 0.828	Z= -2.404 *p* = 0.016* *r* = 0.725	t = 1.579 *p* = 0.153 d = 0.548	Z= -1.450 *p* = 0.147 *r* = 0.548	t = 3.478 *p* = 0.003* d = 0.869	Z= -2.849 *p* = 0.004* *r* = 0.504
Positive Affect	PANAS - Positive	t = 1.917 *p* = 0.077 d = 0.512	Z= -1.639 *p* = 0.101 *r* = 0.438	t = 0.099 *p* = 0.924 d = 0.033	Z= -0.119 *p* = 0.905 *r* = 0.040	n/a	
Negative Affect	PANAS - Negative	t = 3.524 *p* = 0.004* d = 0.942	Z= -2.700 *p* = 0.007* *r* = 0.749	t = 3.623 *p* = 0.007* d = 1.208	Z= -2.314 *p* = 0.021* *r* = 0.771	n/a	
Physical Health (QoL)	SF-12 PCS	t = -0.68 *p* = 0.507	Z= -0.198 *p* = 0.841	t= -0.740 *p* = 0.481	Z= -0.296 *p* = 0.767	n/a	
Mental Health (QoL)	SF-12 MCS	t = -1.52 *p* = 0.146	Z= -1.396 *p* = 0.162	t= -1.210 *p* = 0.261	Z = 1.125 *p* = 0.260	n/a	

PHQ-9 (Patient Health Questionnaire-9), *GADS* (Goldberg Anxiety and Depression Scale), *GAD-7* (Generalized Anxiety Disorder-7), *PANAS* (Positive and Negative Affect Schedule), *SF-12* (Short Form Health Survey-12) - Physical (*PCS*) and Mental (*MCS*) components, *QoL* (Quality of Life), T0 - Assessment at Baseline, T1 - Assessment Post-individual session T2 - Assessment Post-Group Sessions, T1 for the Systemic therapy study was conducted at the end of the intervention (i.e., after 8 online individual sessions, 2 online group sessions and 1 face-to-face group session: ~6 months), * Significant at *p ≤* 0.05

### Findings

Statistical analyses were conducted using paired t-tests and Wilcoxon signed-rank test to compare outcomes across the assessment timepoints.

#### Depression 

Analyses for the individual sessions (T0-T1) showed significant improvement in depressive symptoms across both measures: t = 2.65, *p* = 0.020; Z = − 2.24, *p* = 0.025 for PHQ-9 and t = 2.50, *p* = 0.027; Z = − 2.16, *p* = 0.031 for GADS. At follow-up (T0–T2) after group sessions, the improvement in PHQ-9 scores remained statistically significant (t = − 2.74, *p* = 0.025; Z = 2.40, *p* = 0.016), indicating sustained reductions in depressive symptoms over time. No significant changes were observed for the GADS depression subscale at follow-up (t = 0.94, *p* = 0.376; Z = − 0.64, *p* = 0.523). Our findings were consistent with those observed in the Systemic therapy group, where a significant decrease of depression scores was also found in the non-parametric analysis (Z = − 2.06, *p* = 0.039).

#### Anxiety 

Analyses for the individual sessions (T0–T1) revealed significant reductions in anxiety symptoms. On the GAD-7, scores improved significantly (t = 2.11, *p* = 0.050; Z = − 1.90, *p* = 0.050). Similarly, the GADS anxiety subscale showed significant improvement (t = 3.10, *p* = 0.008; Z = − 2.40, *p* = 0.016). At follow-up (T0–T2), further improvements were observed on the GAD-7 (t = 4.46, *p* = 0.002; Z = − 2.38, *p* = 0.018), indicating sustained reductions in anxiety symptoms. In contrast, no significant differences were observed for the GADS anxiety subscale at follow-up (t = 1.58, *p* = 0.153; Z = − 1.45, *p* = 0.147). These outcomes align with the Systemic therapy group, where the GADS anxiety subscale also reached statistical significance (t = 3.478, *p* = 0.003; Z = − 2.85, *p* = 0.004).

#### Affect

 With regard to affect, no significant changes were observed for positive affect as measured by the PANAS at post- individual sessions (t = 1.92, *p* = 0.077; Z = − 1.64, *p* = 0.101). Similarly, no significant differences were detected at post group sessions (t = 0.10, *p* = 0.924; Z = − 0.12, *p* = 0.905), suggesting that positive affect remained stable across timepoints. In contrast, negative affect showed a significant reduction following the individual sessions (t = 3.52, *p* = 0.004; Z = − 2.70, *p* = 0.007), reflecting a decrease in the frequency of negative emotional states (e.g., irritability, nervousness, guilt). These improvements remained significant after group sessions (t = 3.62, *p* = 0.007; Z = − 2.31, *p* = 0.021), indicating a sustained reduction in negative affect over time.

#### Quality of life 

No significant changes were observed in quality-of-life outcomes for either the physical component of the SF-12 (t = − 0.68, *p* = 0.507; Z = − 0.20, *p* = 0.841) or the mental component (t = − 1.52, *p* = 0.146; Z = − 1.40, *p* = 0.162) for the individual sessions (T0–T1). Similarly, no statistically significant changes were found after group sessions (t = − 0.74, *p* = 0.481; Z = − 0.30, *p* = 0.767 for the physical component; t = − 1.21, *p* = 0.261; Z = 1.13, *p* = 0.260 for the mental component).

#### Effect sizes 

The observed effect sizes suggest meaningful improvements in several psychological outcomes. For *depression*, medium-to-large effects were observed following the individual sessions (T0–T1) (d = 0.71, *r* = 0.65 for PHQ-9; d = 0.67, *r* = 0.60 for GADS). These improvements were largely maintained after the group sessions (T0–T2), with large effects on the PHQ-9 (d = 0.75, *r* = 0.91) and small effects on the GADS depression subscale (d = 0.31, *r* = 0.22); our data are consistent with clinical improvements and in line with the Systemic therapy group (d = 0.52, *r* = 0.37).

Similarly, for *anxiety*, moderate-to-large effects were observed after the individual sessions (d = 0.56, *r* = 0.53 for GAD-7; d = 0.83, *r* = 0.73 for the GADS anxiety subscale). At follow-up, effect sizes indicated a large, sustained improvement on the GAD-7 (d = 1.49, *r* = 0.90) and moderate effects for the GADS anxiety subscale (d = 0.55, *r* = 0.55); our data are consistent with the findings in the Systemic therapy study (d = 0.87, *r* = 0.50). Regarding affect, the *PANAS positive* subscale showed a moderate effect after the individual sessions (d = 0.51; *r* = 0.44) despite non-significance, but negligible change after the group sessions (d = 0.03; *r* = 0.04). In contrast, the *PANAS negative* subscale demonstrated large effects at both timepoints (d = 0.94; *r* = 0.75 at T0–T1; d = 1.21; *r* = 0.77 at T0–T2), reflecting a substantial and sustained reduction in negative affect. Taken together, these findings suggest that the intervention had its strongest impact on mood-related outcomes (depression, anxiety, negative affect), whereas perceived quality of life remained largely unchanged.

## Discussion

This study explored the impact of a cognitive-behavioural therapy intervention on mood, anxiety and quality of life in individuals at risk of HD, with premanifest HD, and with a negative genetic test result. To our knowledge, this is the first study to address the needs of all three groups—at-risk, premanifest, and negative-test individuals—within a single intervention framework. Moreover, the prospective design and the validated outcome measures used, further enhance the robustness of the findings. Given the limited empirical evidence available to support the efficacy of psychological interventions, our study makes a valuable contribution to the literature. A systematic scoping review by Zarotti et al. (2020) highlighted significant gaps in the literature, especially in terms of methodology and clinical outcomes. There seems to be an “alarming lack of data on the subject and a consequent scarcity of dedicated psychological services” (Zarotti et al. 2024) to individuals and families impacted by HD. A single case study by Silver (2003) reported the positive impact of a CBT-based intervention in reducing both the anxiety and depression levels of an individual with premanifest HD. A’Campo et al. (2012) explored the effectiveness of a Participant Education Program for HD, which combined psychoeducation with CBT components, on anxiety, depression, and coping skills of individuals with symptomatic and premanifest HD. The results showed significant improvements in depression and specific components of coping (e.g., seeking social support and reduced use of passive reactions) for both premanifest and symptomatic participants. Based on a large longitudinal non interventional studies with a significant number of individuals with premanifest HD (*n* = 192), Boileau et al. (2020) suggested that participants frequently expressed the need for greater emotional support and counselling to help manage the emotional changes associated with HD and to reduce feelings of stigma. A recent study by van Lonkhuizen et al. (2024) showed that HD gene expansion carriers might benefit from forming helpful beliefs about future adaptability and resilience and developing adaptive coping strategies. Together, these findings suggest a strong perceived need for targeted psychological interventions to enhance emotional support and coping in individuals impacted by HD.

Our study demonstrated that an eight session CBT intervention produces meaningful improvements in depression, anxiety, and negative affect. This reduction is particularly noteworthy, as emotional dysregulation is a common and distressing feature of HD, impacting both individuals affected by HD and their families. We did not identify any significant changes in quality-of-life suggesting that this concept is likely to be less sensitive to short-term psychological interventions. The SF-12 scale captures broad aspects of physical and mental health functioning, which typically require longer periods or more intensive multidisciplinary interventions to show measurable change. This is in line with previous findings where the sensitivity of the instrument is less certain longitudinally (Ho et al. 2009). Taken together, the moderate-to-large effect sizes observed for depression, anxiety, and negative affect underscore that the intervention produced meaningful changes, even when statistical significance was limited by the small sample size. These findings highlight the value of CBT-informed approaches in addressing mood and affective symptoms in individuals impacted by HD, while also pointing to the need for longer-term or more intensive interventions to meaningfully influence perceived quality of life. Because this was an uncontrolled, small sample size study, we cannot attribute observed changes solely to the intervention; improvements may reflect participant expectancy or natural fluctuations in psychological adjustment over time. Without comparison to a control group or to known natural trajectories of psychological adjustment in HD, causal inference is limited and results should be interpreted as preliminary evidence warranting controlled, longitudinal replication.

Our findings complement and extend previous research on psychological interventions in HD. Previous studies have reported improvements in depression, anxiety, and coping skills for specific participant groups, using different intervention modalities (Silver 2003; A’Campo et al. 2012; Stopford et al. 2019; MacLeod et al. 2018; Spiers et al. 2020). Our study demonstrates that a single, integrated intervention can produce meaningful and sustained reductions in depressive and anxiety symptoms. In this way, our data help fill gaps identified in prior reviews (Zarotti et al. 2020), showing consistent benefits across a broader spectrum of individuals and psychological outcomes, while also providing effect-size estimates to quantify the magnitude of these improvements.

The delivery of psychological interventions in HD requires clinicians with formal training in CBT psychotherapy, as well as specialist knowledge of HD and the psychosocial consequences across the disease spectrum. In this study, both the clinical psychologists (SS and MR) and assessor (ST) were certified in CBT psychotherapy and had extensive HD-specific training, together with basic training in genetic counselling, which likely supported the adaptation of the intervention to the uncertainty, family context, and emotional complexity of HD. Because of the particularities of this condition, future psychological interventions for HD should ideally be delivered by professionals with dedicated HD training, such as the educational pathway offered through the Huntington Academy program of the European Huntington Association (European Huntington Association, n.d.).

Several limitations of the present study warrant consideration. First, the small sample size and exploratory nature of the analyses suggest caution in generalising the findings. Moreover, the absence of a control group limits the ability to draw definitive causal inferences regarding the observed changes. Another methodological consideration is the difference in the design of the two studies (e.g., assessment time points, measurement instruments, eligibility for participants), which complicate direct comparisons. Accordingly, such comparisons should be interpreted with caution, although the consistency in the direction of effects and the similarity of the observed benefits across groups are noteworthy. Another factor that needs to be considered when interpreting results is clinical heterogeneity. To maximize accessibility, no eligibility criteria related to participants’ well-being at enrolment were imposed, resulting in a sample with considerable variability in baseline characteristics. While this approach enhanced ecological validity of our results, it also led to the exclusion of three participants due to specific circumstances. Although these exclusions were methodologically justified, they further reduced the statistical power and highlight the importance of structured eligibility screening in future research. Taken together, these limitations emphasize the need for adequately powered studies with clearer inclusion criteria, larger sample sizes, and randomized controlled designs. Future research should seek to replicate and extend these findings to establish the efficacy of psychological interventions for HD populations. Such studies should also examine long-term outcomes through multiple follow-up assessments, and explore potential moderators of treatment response, such as genetic status, disease stage, and baseline psychosocial functioning. Addressing these factors will help identify which individuals are most likely to benefit and inform the development of tailored intervention strategies.

This study provides evidence supporting the efficacy of a CBT intervention in reducing depression, anxiety, and negative affect among individuals at risk of HD, those with premanifest HD, and individuals with a negative genetic test result. Although no significant improvements were observed in quality of life or positive affect, the consistent moderate-to-large effect sizes for mood-related outcomes highlight the potential value of tailored psychological support as an integral component of comprehensive HD care. While further research is needed to replicate and expand these findings, and address the study’s limitations, the present results highlight the importance of incorporating psychological interventions into standard HD care. By addressing the psychological burden faced by individuals affected by HD and their families, such interventions hold considerable promise for improving overall well-being. These findings provide a compelling rationale to advocate for earlier and more universally accessible mental health care across the HD spectrum.

## Data Availability

Anonymised data is available upon reasonable request from the corresponding author.
